# Deep learning for the quality control of thermoforming food packages

**DOI:** 10.1038/s41598-021-01254-x

**Published:** 2021-11-08

**Authors:** Núria Banús, Imma Boada, Pau Xiberta, Pol Toldrà, Narcís Bustins

**Affiliations:** 1 Graphics and Imaging Laboratory, University of Girona, 17003 Girona, Catalonia Spain; 2 Vision Department (R&D), TAVIL Ind. S.A.U., 17854 Girona, Catalonia Spain

**Keywords:** Engineering, Computer science, Information technology, Software

## Abstract

Quality control is a key process designed to ensure that only products satisfying the defined quality requirements reach the end consumer or the next step in a production line. In the food industry, in the packaging step, there are many products that are still evaluated by human operators. To automate the process and improve efficiency and effectiveness, computer vision and artificial intelligence techniques can be applied. This automation is challenging since specific strategies designed according to the application scenario are required. Focusing on the quality control of the sealing and closure of matrix-shaped thermoforming food packages, the aim of the article is to propose a deep-learning-based solution designed to automatically perform the quality control while satisfying production cadence and ensuring 100% inline inspection of the products. Particularly, the designed computer vision system and the image-based criteria defined to determine when a product has to be accepted or rejected are presented. In addition, the vision control software is described with special emphasis on the different convolutional neural network (CNN) architectures that have been considered (ResNet18, ResNet50, Vgg19 and DenseNet161, non-pre-trained and pre-trained on ImageNet) and on the specifically designed dataset. To test the solution, different experiments are carried out in the laboratory and also in a real scenario, concluding that the proposed CNN-based approach improves the efficiency and security of the quality control process. Optimal results are obtained with the pre-trained DenseNet161, achieving false positive rates that range from 0.03 to 0.30% and false negative rates that range from 0 to 0.07%, with a rejection rate between 0.64 and 5.09% of production, and being able to detect at least 99.93% of the sealing defects that occur in any production. The modular design of our solution as well as the provided description allow it to adapt to similar scenarios and to new deep-learning models to prevent the arrival of faulty products to end consumers by removing them from the automated production line.

## Introduction

The Industry 4.0 paradigm is changing the companies’ layout towards automated production processes^1^. Manual procedures are replaced by automated production lines where different machines perform specific operations to transform raw material into final products. As a result, more efficient and effective processes are achieved^2–4^. Although the configuration of the production lines depends on the complexity of the manufacturing parts, they usually include a packaging stage. Packaging is not only related to product protection for storage, shipping and sale, but is also used to provide information to the user as well as to promote the product through marketing strategies^5^.

The importance of packaging has led industries to look for continuous improvements in production methods as well as in materials, with emphasis on the principles and applications of active and intelligent packaging, nanotechnology, antimicrobial packaging, edible coatings and films, and sustainable packaging. In the context of active packaging, for instance, Jariyasakoolroj et al.^6^ reviewed the recent developments in bioplastic food packaging, emphasizing the advances in applications requiring gas and water barrier; Srisa and Harnkarnsujarit^7^ investigated the impact of antifungal film properties for bread packaging; Khumkomgool et al.^8^ studied the effect of incorporating herbal extracts into films to produce functional active packaging and preserve the qualities of packaged meat products; Chatkitanan and Harnkarnsujarit^9^ evaluated the quality of pork when nitrite is incorporated into the active films used for its packaging; and Laorenza and Harnkarnsujarit^10^ investigated bioplastic films containing essential oils.

One of the key processes of the packaging stage is the quality control that inspects the packed product to ensure it satisfies the quality requirements before reaching the final user. The application of computer vision and artificial intelligence techniques allows this process to be done automatically. Medus et al. (2021)^11^, for instance, proposed a system which, by using a convolutional neural network (CNN) as a classifier in heat-sealed food trays, is able to automatically detect anomalies during the packaging process in order to discard the faulty tray and avoid human consumption. Thota et al. (2020)^12^ presented a multi-source deep-learning-based domain adaptation system to identify and verify the presence and legibility of use-by date information from food packaging photos taken as part of the validation process as the products pass along the food production line. Brunelli et al. (2019)^13^ designed a deep-learning-based approach for production performance forecasting in fresh products packaging. As these, many other solutions can be found in the literature, all of them with the common feature that they have been specifically designed to meet the requirements of the evaluated product and also the conditions of the environment where they have to be installed^14–19^. Therefore, the design of such solutions, and particularly the CNN, is one of the more challenging tasks for the automation of the quality control processes.

The design of a CNN-based approach requires a training process to optimize the large set of CNN parameters. This process is computationally intensive, since a large amount of labeled visual data has to be passed through the CNN layers to ensure high levels of accuracy in regression and classification tasks^20, 21^. However, if transfer learning is used, both training time and computational cost can be reduced^22^. Transfer learning is a machine learning technique that exploits the fact that the early layers of a CNN learn low-level features, which are applicable to most computer vision tasks, while the subsequent layers learn high-level features, which are mostly application-specific. In this way, transfer learning considers a pre-trained CNN and re-trains a subset of its parameters by using a smaller amount of data to perform well on a new task. Consequently, the preparation of this domain-specific dataset becomes a challenge^23^. Although different networks^24^ including AlexNet^25^, ResNet^26^, Vgg^27^ and DenseNet^28^, pre-trained with datasets such as the ImageNet^29, 30^, are available, it remains to be determined which of them performs best in a specific scenario.

Taking into account all these considerations, the company involved in this study, which is specialized in the construction of machinery and automatic lines for packing, palletizing and handling, was interested in the development of a CNN-based solution for the automatic quality control of food packages. After evaluating different off-the-shelf solutions^31, 32^ it decided to develop an in-house product to reduce the shortcomings of proprietary systems that limit the customization capabilities of the solution, since the creation of these new functionalities requires an extra cost for the company. At the expense of extra time for the software development, the in-house solution will make the integration of new functionalities and the adaption to new scenarios easier. Moreover, different software frameworks and open-source libraries that include state-of-the-art image processing techniques and deep-learning algorithms are available, thus making the development process less difficult^31–37^.

For the development of this solution, the quality control of thermoforming food packages of a real company has been considered. The thermoforming process places the product in a tray or a preformed packaging mould that is then sealed with skin film. Since different forms of packaging can be produced^4^, the complexity of the automation of the quality control process will depend on the product shape. In this paper, we are going to consider products with a matrix shape that also require a cutting step to obtain single- or multi-pack final configurations^38^. The quality control performs a serigraphy test that checks the correct position of the printings, a traceability test that checks the good visibility of the date and the lot number, and a closure and sealing test. We will center on this last test, as it is the most critical one^39^. The sealing preserves the product while maintaining its properties, thus having a direct impact on its quality. Faults on the sealing will lead to non-consumable products, and these have to be removed from the production line. Material properties such as transparency or serigraphy make the detection of sealing faults via computer vision systems difficult. Several systems have been proposed to classify, control, or identify sealing defects by using polarized light stress analysis and laser scatter imaging^40^, active infrared thermography^41^ or hyperspectral imaging^11^.

Based on this practical case, the aim of this paper is two-fold. On the one hand, it will propose a deep-learning-based solution to automatically perform the quality control of the sealing of matrix-shaped thermoforming food packages while satisfying production cadence and ensuring 100% inline inspection. The proposed solution will be tested and evaluated in the laboratory and also in a real scenario. On the other hand, it will present a comparison of different deep-learning classification models^26–28^ and also different specifically designed datasets; a comparison that will be useful for deciding the best model in similar situations. Although the proposed solution will be presented in the context of a specific scenario, the solution can be extended to other scenarios where other deep-learning-based solutions are to be applied and other computer vision strategies are required. The modular design of our proposal as well as the provided description make the adaption to new scenarios and to new deep-learning models straightforward, since the basis of the approach is always the same.

In Table 1 our proposal is compared with some of the systems previously described considering: the target where the quality control approach is applied; the classification accuracy obtained; the method applied; the extracted features; the input data required by the method; the datasets used for training, testing, and validation, and whether or not data augmentation has been applied; and the scenario in which the method has been tested.

**Table 1:** Comparison of some state-of-the-art methods, including our proposal in the last column. *SVM* = Support Vector Machine, *RBF* = Radial Basis Function, *CNN* = Convolutional Neural Network, *Pre* = Pre-trained with ImageNet, *HSI* = Hyperspectral Imaging, *PL* = Polarized, *LS* = Laser Scatter, *ch* = channels, *f* = features, *Mono* = Monochrome, and *IRAS* = Infrared image Acquisition System.

	Medus et al.^11^	Thota et al.^12^	Prada-López et al.^17^	Xie et al.^18^	Barnes et al.^40^	Our method
**Quality control**	Sealing of heat-sealed food trays (Anomalies detected: washers, sugar, flange, plywood, cork, elastic rubber, wood, paper, sliver paper, hair, and polarized plastic)	Presence and legibility of use-by date information from food packages	Types of coffee and adulterations	Atlantic salmon bone residues	Detect copper wire in the seal of heat-sealed packages	Sealing of thermoforming food packages
**Method (Accuracy)** SVM RBF	84.3%					
CNN-ResNet18						96%
CNN-ResNet18(Pre)						96%
CNN-ResNet50	95%					94%
CNN-ResNet50(Pre)		92.5%				97%
CNN-ResNet34(Pre)			98.6%			
CNN-VGG16				87%		
CNN-VGG19				86%		93%
CNN-VGG19(Pre)						98%
CNN-AlexNet				76%		
CNN-DenseNet161						98%
CNN-DenseNet161(Pre)						**99%**
Real AdaBoost					96% PL; 90% LS	
**Feature extraction**	HypLabTool. 10, 50, and 130 spectral channels for each ROI 40 x 40 (one single ROI at the same time)	Multi-source domain adaptation	Raw image	Raw image with different compression ratio and Faster-RCNN	Features per channel of 16 regions of interest of the seal	**Domain-specific dataset evaluation**
**Input data**	HSI images	RGB images	RGB images	RGB images	PL (70ch; 420f) and LS (20ch; 120f) images	**Mono. images IRAS**
**Datasets**	Real	Real	Intentional	Real	Intentional	Real
Total	2248 ROIs	30000	6237	260	117	3606
Training	1348	21000	4678	208		2978
Test	562	6000	312	52		**Real scenario**
Validation	338	3000	1247		Hold-one-out	628
Data augmentation	No	No	Yes	Yes	No	No
**Testing scenario**						
Laboratory	Yes	Yes	Yes	Yes	Yes	Yes
Real	Yes	No	No	Yes	No	**Yes**

## Materials and methods

This section describes the details of the proposed solution focusing on the hardware and software components, and paying special attention to the CNN approach.

### Image acquisition system

**Figure 1:**
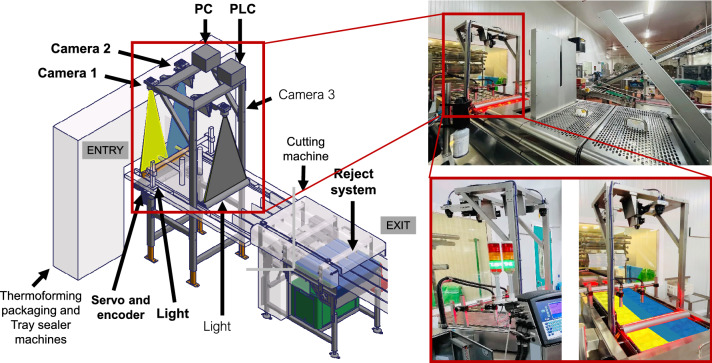
A schema of the main components of the proposed computer vision system (highlighted in bold) with some views of the real scenario. The cameras are placed between the tray sealer and cutting machinery; a servo and an encoder, connected to a Programmable Logic Controller (PLC), control the speed of food packages movement, the position of the products, and when the reject system has to act. Monochrome line scan *Camera* 1 and *Camera* 2 are triggered with different exposure times to acquire images for the sealing test. *Camera* 3 acquires images for the serigraphy and traceability tests. Then, the acquired images are processed to determine how the reject system has to proceed.

Figure 1 depicts the packaging phase of the food company considered in the real scenario. This phase involves the thermoforming packaging machine, which provides the lower part of the package with multiple cavities that will contain the food; the tray sealer machine, which seals the upper film on the filled food containers; and the cutting machine, which cuts the multiple containers according to the needs of the brand in single or double (bi-pack) packages. The transition between machines is set according to the production cadence and ranges from 6 to 8 steps per minute with steps of 700 mm height and 525 mm width moving at 0.7 m/s. The cycle time of one step is 10 s, in 9 of which packaging tasks are performed.

To automate the quality control process, the first two issues that are addressed are (i) the computer vision system that will be used to obtain the images of the products, and (ii) the features of the images that have to be evaluated to determine whether a product has to be accepted or rejected.

#### Computer vision system

The main components of the proposed computer vision system are depicted in Fig. 1. The system has three cameras, one to perform the serigraphy and traceability tests, and the other two for the closure and sealing test. Our interest is focused only on the latter: two 4K monochrome CMOS line scan cameras with an Infrared Bandpass Filter at 850 nm, standard GigE Vision interface (80 Mb/s)^42^ and TurboDrive technology^43^. A linear IR 850 nm led light with a diffusion filter positioned behind the product acts as a backlight. The products move perpendicular to the fixed acquisition system, which obtains images of 4096 pixels (4K) with a $7.04 \times 7.04$ µm pixel size.

The features of the products to be processed as well as the configuration of the company’s production line are what determine the positions of the camera and the illumination system. Particularly, the main factors that have been taken into account are: (1) the stability of the system to avoid the vibrations caused by other machines of the same production line; (2) the space required by human operators to perform different procedures such as maintenance tasks; and (3) the information acquired by the cameras to ensure that all the features that have to be examined to determine whether the product has to be accepted or not are visible in the images. Driven by these restrictions and after testing with different products and configurations, the final setup is presented below.

The system has been installed between the tray sealer and the cutting machinery, as illustrated in Fig. 1. This is the location that meets the space and cadence restrictions for the current machines layout. The cameras have been placed at a height of 1015 mm with respect to the products to ensure the stability of the system in case of vibrations caused by other machines of the production line, and also to ensure the human operability required in different procedures such as maintenance tasks. The width of the field of view (FV) of each camera is 270 mm, ensuring that the entire products’ width is covered in the images. The light has been placed at a height of 110 mm below the products to reach the point where the camera focuses. The light covers a width of 700 mm to ensure the illumination of the whole area and the correct detection of the features. Such a configuration provides a complete view of the packages (single- or bi-pack products, depending on whether one or two cavities are taken) in the images. In addition, the two-camera configuration reduces possible deformations or undesired shadows that can appear in the products placed at the borders of the image. Moreover, its high speed also fits the cadence requirements.

**Figure 2:**
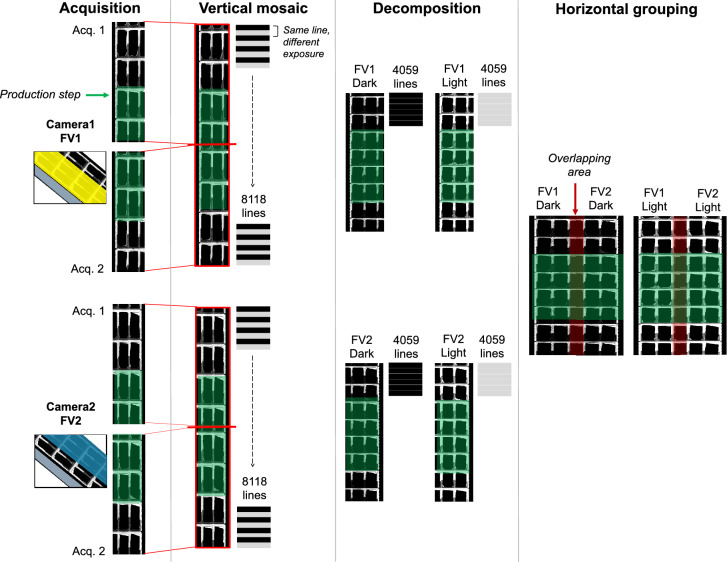
Sequence of images obtained during the acquisition process. From left to right: *Acquisition* contains images of two consecutive steps from *Camera*1 (FV1) and *Camera*2 (FV2) that together cover the whole field of view (FV); *Vertical mosaic* contains images from the vertical combination of the two consecutive acquired images; *Decomposition* contains images obtained from the division of the combined image into two images with different exposure; and *Horizontal grouping* contains the final images with different exposure that will be analyzed.

In Fig. 2, the different images that are involved in the acquisition process are presented. The line scan technology used^44^ acquires two-dimensional images line by line while the products move perpendicular to the fixed acquisition system. Since the installation of the computer vision system depends on the packaging machines layout, it may happen that the camera’s field of view does not cover all the products in a single production step. This situation is represented in Fig. 2, where images are acquired by two cameras (labelled as *Camera*1 and *Camera*2) with their different fields of view (FV1 and FV2). The first step, *Acquisition*, presents the images acquired by Camera1 and Camera2, which cover the yellow and blue parts, respectively. Each camera performs two consecutive acquisitions, covering all four rows of products when vertically combined in *Vertical mosaic*. As mentioned, this is necessary because the position of the system does not allow the cameras to capture all the rows in a single production step. To obtain all the relevant information, each line of the image is acquired twice for each encoder pulse, with each copy having a different exposure. The vertically combined image contains 8118 lines, which in the *Decomposition* step are divided into two images with 4059 lines, one with dark lines and the other with light lines. The last step, *Horizontal grouping*, combines the light and dark images from each field of view, resulting in images that provide two full views of the products with two different exposures.

#### Accept/reject criteria

**Figure 3:**
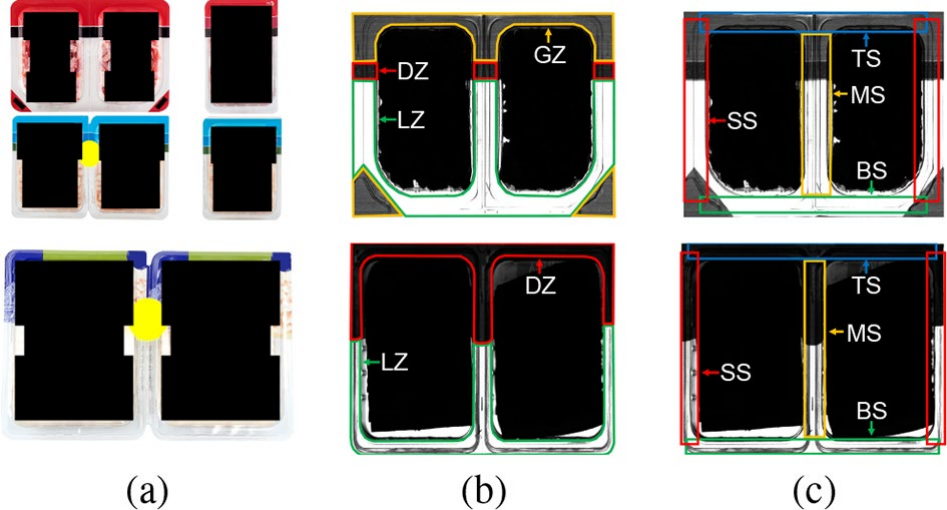
Sealing zones for all products: (**a**) products of different brands in bi-pack and single format; (**b**) light (LZ), dark (DZ) and gray zones (GZ), common to all packages; and (**c**) sealing zones according to the position, including top (TS), bottom (BS), side (SS) and middle sealing (MS).

The accept/reject criteria have to be defined on the basis of the information that can be obtained from the acquired images. As depicted in Fig. 3(a), the size, the shape and the distribution of opaque and transparent areas of the products as well as the color of the film are different depending on the brand. Despite these differences, there are some features in the sealing area that are common to all of them. Particularly, the sealing area of the products can be divided into: light zones (LZ), which have a transparent and constant film; dark zones (DZ), which have a different colored area in the film and appear in dark gray in the images; and gray zones (GZ), which appear in light gray in the images (see Fig. 3b). These differences can be processed by the installed computer vision system. In addition, there are other zones of the sealing that can be defined according to the position, both for the single and bi-pack configurations of the final packages. These zones are presented in Fig. 3(c) and correspond to top sealing (TS), bottom sealing (BS), side sealing (SS), and middle sealing (MS), the latter appearing only in bi-pack packages.

**Figure 4:**
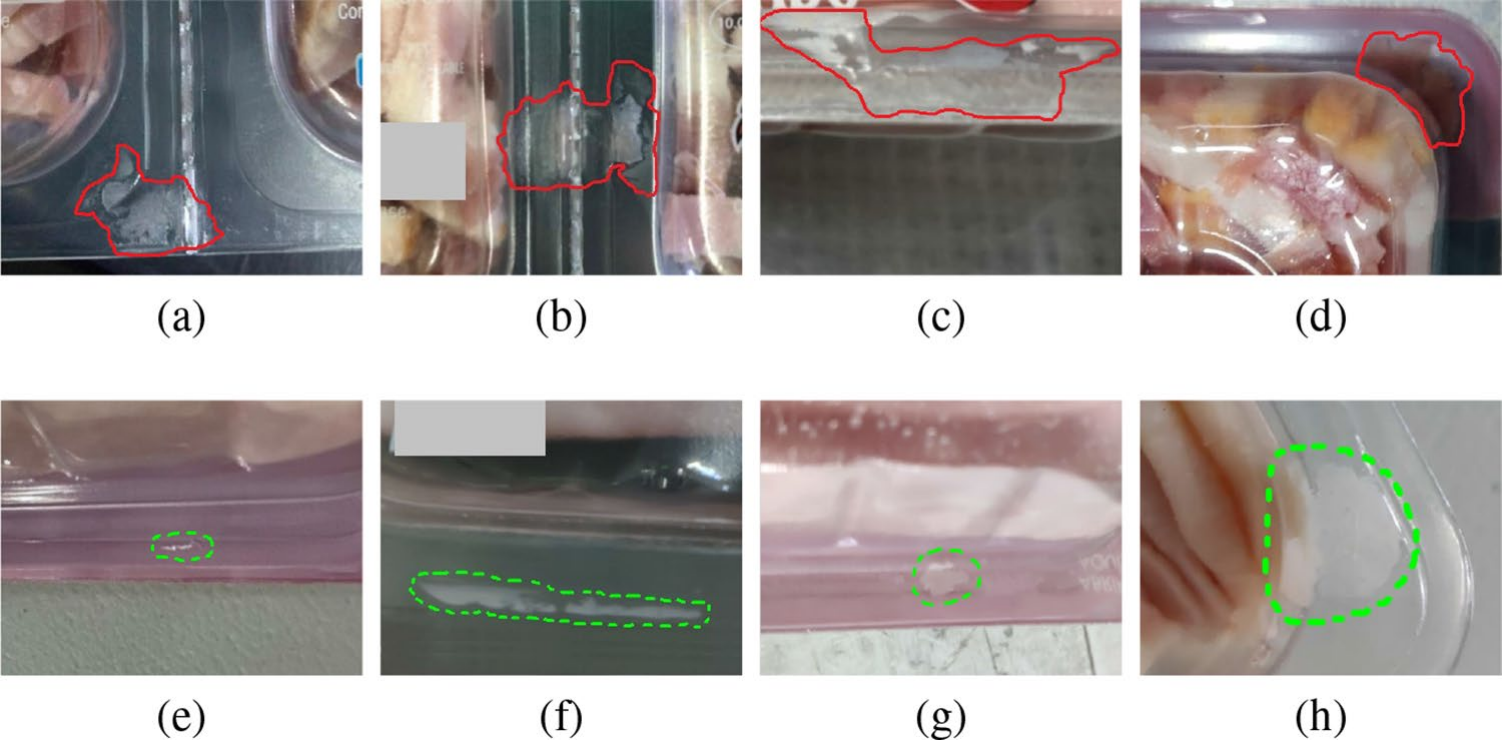
Examples of (**a**–**d**) reject and (**e**–**f**) accept cases where the relevant areas have been outlined in red and green, respectively.

Moreover, to identify the causes that determine the acceptance or rejection of a product, the employees that currently perform the quality control of the products were observed. Besides rejection due to failing the serigraphy or traceability tests, it was observed that a food package is normally rejected if at least one of the following conditions is given: (i) there is a large piece of food or a bubble passing through the sealing (see Fig. 4a–c); or (ii) there is a large piece of food near the sealing that, despite not being in the sealing, will have an undesired appearance to the end user (see Fig. 4d). On the other hand, there are other situations that are not a reason for rejection, such as: (i) the marks of the machine that joins the film with the product (see Fig. 4e); (ii) the moisture generated by the temperature of the food at sealing time (see Fig. 4f); (iii) the small pieces of food in the package sealing that do not pass through it (see Fig. 4g); and (iv) the small or large pieces of food near the package sealing that do not have an undesired appearance (see Fig. 4h).

### Control vision software

The control vision software determines when a product has to be accepted or rejected. The key component of this software will be the CNN, which will require a proper dataset to train, validate, and test it using data collected in production. Therefore, the next two issues to be addressed are (i) the definition of the domain-specific dataset required for the training, and (ii) the selection of the CNN that will best fit our scenario. In addition, a global view of the main modules of the software will be provided.

#### Domain dataset preparation

**Figure 5:**
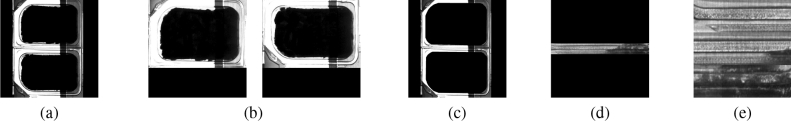
Dataset definition considering the product-based approach and focusing on (**a**) the whole bi-pack product, (**b**) single packs by means of a bi-pack to single pack division, and (**c**) the whole product with a sealing mask; and considering the sealing-based approach and focusing only on (**d**) the pixels in the sealing area (including irrelevant black pixels), and (**e**) the sealing parts obtained after a cutting process and grouped as a sealing mosaic.

To prepare the domain-specific dataset, two different sets of images acquired with the proposed vision system have been considered (see Fig. 2). The first one, $S_1$, has been obtained from the division of 400 acquired images (200 with each type of exposure) into images with a single bi-pack package (see Fig. 5a). This set has 4465 images, which have been labeled as accepted (3805) or rejected (660), and which have then been distributed in a training (80%) and in a validation (20%) dataset. The second one, $S_2$, has been obtained from the division of a subset of acquired images into new images where only the sealing region is represented (see Fig. 5d). Again, the images have been labeled as accepted (390) or rejected (160), and then distributed in a training (80%) and in a validation (20%) dataset. Note that a large number of images is not required for a first evaluation and no testing dataset will be considered, since the testing will be done with the real data from the industrial scenario. From these images, five different domain-specific datasets have been created.

The first three datasets have been obtained from $S_1$, applying a product-based approach with three different strategies. The first one, named *whole bi-pack product*, considers $S_1$ images scaled to $224 \times 224$ pixels by using the nearest neighbor interpolation and normalizing the pixel values (see Fig. 5a). The second one, named *bi-pack to single pack division*, divides the bi-pack product represented in $S_1$ images into two single packs, obtaining 8816 images that are labeled as accepted (8131) or rejected (685) (see Fig. 5b). With this strategy, not all images can be split correctly, thus losing some $S_1$ data. The third one, named *sealing mask*, focuses only on the sealing of the package by removing the internal part with a mask (see Fig. 5c). In all cases, the obtained images are scaled to $224 \times 224$ pixels and the pixel values are normalized as in the first case.

The last two datasets have been obtained from $S_2$, applying a sealing-based approach with two different strategies. The first one, named *only sealing*, considers only the sealing parts, and scales up images to $512 \times 512$ pixels by using the nearest neighbor interpolation and normalizing the pixel values (see Fig. 5d). In this case, downscaling is not possible since important information could be lost. The second one, named *sealing mosaic*, generates a new image where only sealing parts are represented. These parts are cut from the above dataset and represented one below the other (see Fig. 5e). New images have $201 \times 201$ pixels and no resizing has been required. This is the only case where the processed information has the actual dimensions of the original images.

To prepare the datasets, no data augmentation process has been required. Note that our system acquires images with two exposures, thus obtaining a large number of images with different light intensities. Transformations such as horizontal and vertical flipping or some rotations are not suitable due to the nature of the acquisition process, which always has the same position and movement with respect to the vision system. Zooming in or random lighting strategies are not appropriate either, since they could lead to loss of information with an undesired effect on the dataset.

The five different domain-specific datasets that have been created will be evaluated in “Domain-specific dataset evaluation” section to determine which one is best for preparing the dataset for the real scenario.

#### Convolutional neural network

In this study, we considered three deep-learning architectures, namely ResNet, Vgg and DenseNet, which are commonly used for image classification^45^. ResNet, proposed by He et al. (2015)^26^, is characterized by the residual block and skip connections between blocks. This architecture alleviates the problem of gradient disappearance caused by increasing the network depth, and also improves accuracy by adding considerable depth. In our case, we are going to consider ResNet18, with 18 layers, and ResNet50, with 50 layers. Vgg, proposed by the Visual Geometry Group at Oxford University^27^, is a classic convolutional neural network designed to enhance the classification accuracy by increasing the depth of the network. The two commonly used models are Vgg16 and Vgg19, with 16 and 19 layers, respectively. In our case, we are going to consider Vgg19. Finally, DenseNet, proposed by Huang et al. (2018)^28^, is a densely connected convolutional network. In this model, the input of each layer comes from the output of all the previous layers, establishing connections between them. It can alleviate the problem of gradient disappearance, strengthen the feature transmission, reduce the number of parameters and enhance effective feature utilization. There are four usual network models according to the number of layers: DenseNet121, DenseNet169, DesNet201 and DenseNet161. In this study, we are going to consider DenseNet161.

The models will be evaluated with and without pre-training on ImageNet^46^. Pre-trained models require input images to be equally normalized with 3 channels and pixel values in the range [0, 1]. The training will be done using the Python programming language^47^ by means of the PyTorch^36^ and FastAI^33^ optimized tensor libraries for deep learning, and considering a variable learning rate by using the optimizer based on the Adam’s algorithm^48, 49^. The loss function used combines a Sigmoid layer and the Binary Cross-Entropy Loss (BCELoss) into a single class^50^. For pre-trained models, a two-step training will be carried out: the first two epochs will run with frozen layers, while the next ones will run after the layers have been unfrozen. An Intel(R) Core(TM) i7-7800X CPU (64 RAM) with one NVIDIA GeForce RTX 2080 Ti (64 GB of RAM) will be used.

To evaluate the CNN performance, the standard derived measures (see Table 2) will be used, including *accuracy* (A), the ratio of correct predictions to the total number of predictions, *precision* (P), the ratio of correct positive predictions to the total number of positive predictions, *recall* (R), the ratio of predicted positives to the total number of positive labels, and *F-score* (F), the harmonic mean of precision and recall.

**Table 2:** Formulas for the statistical features extracted from the confusion matrix, where $n_{TP}$, $n_{FP}$, $n_{TN}$ and $n_{FN}$ represent the number of true positives, false positives, true negatives and false negatives, respectively.

Measure	Formula
Accuracy (A)	$\dfrac{n_{TP} + n_{TN}}{n_{TP} + n_{TN} + n_{FP} + n_{FN}}$
Precision (P)	$\dfrac{n_{TP}}{n_{TP} + n_{FP}}$
Recall (R)	$\dfrac{n_{TP}}{n_{TP} + n_{FN}}$
F-score (F)	$2 \times \dfrac{Precision \times Recall}{Precision + Recall}$
False omission rate (FOR)	$\dfrac{n_{FN}}{n_{FN} + n_{TN}}$
False discovery rate (FDR)	$\dfrac{n_{FP}}{n_{FP} + n_{TP}}$

We will also compute the *false omission rate* (FOR) to measure the products with a defect that are not rejected and that continue on the production line as if they were correct products, and the *false discovery rate* (FDR) to measure the products that have been rejected when they should have been accepted. Both metrics can be used to evaluate the CNN model depending on the context needs. If health security is a critical issue, the FOR measure will be the main indicator; if the number of correct products classified as faulty needs to be reduced, the FDR measure will need to be low.

#### Integration of the CNN-based solutions into a real scenario

The integration of CNN-based solutions into real scenarios is complex, since these solutions have been developed in the context of intricate frameworks with high performance capabilities using both CPU and GPU, which is not common in industrial scenarios. To overcome this limitation, our approach proposes a modular design based on an optimized C# library that allows different classification algorithms to be executed and is also able to integrate into different hardware platforms to fit the industrial needs.

**Figure 6:**
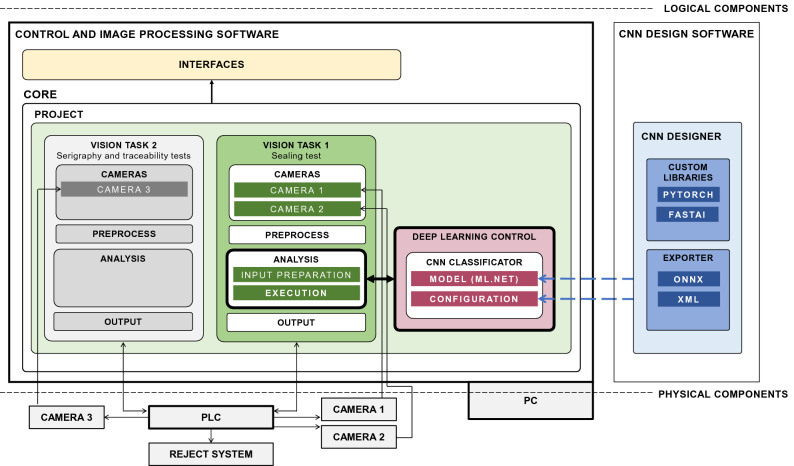
Main modules of the proposed software (example).

**Figure 7:**
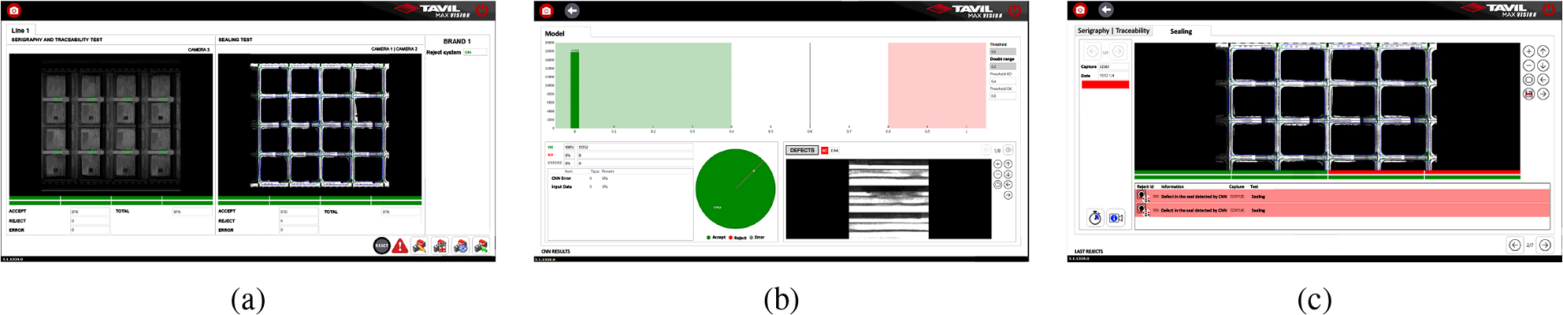
Interfaces of the control and image processing software with (**a**) the main window of the software, which shows the production in real time, the test results for each analyzed step (the serigraphy and traceability test on the left and the sealing test on the right), and the total counters; (**b**) the CNN Inference Statistics window, which shows the last input images labeled as rejected by the model, the total counters, the decision threshold and doubt range, and the graphically represented historical probability; and (**c**) the Last Rejected Steps window, which shows the result of the last steps rejected by a test.

The main components of the software that controls the computer vision system are presented in Fig. 6. The logical components are the *Control and Image processing Software* and the *CNN Design Software*. The first component is an in-house product designed to manage computer vision systems of a company^51^. It is composed of: (i) *Interfaces*, with functionalities for the user to interact, modify parameters of the project that is running, and also visualize related information (see Fig. 7); and (ii) the *Core*, with a *Project* module that defines and manages the components according to the product being processed. The Project determines how the control vision software has to proceed via *Vision Tasks*. In this example, there are two Vision Tasks, one to control the serigraphy and traceability tests, and the other one to control the sealing test. The components of a Vision Task are: (i) the *Cameras*, either one or more working together ; (ii) the *Preprocess*, which transforms acquired images into images ready for processing (see Fig. 2); (iii) the *Analysis*, which applies different computer vision techniques to analyze images according to case requirements; and (iv) the *Output*, which returns the final result to indicate to the PLC how the reject system has to proceed. The PLC also indicates when related tasks have to start. The Analysis performs an *Input preparation* step to prepare the images to be analyzed so that they fit into the next step (for instance, in the sealing test it prepares the images for the inference process), and an *Execution* step to carry out the required procedure, which may be deterministic or, as in the case of the sealing test, the inference of the model.

The *CNN Design Software* component is connected to the Control and Image processing Software at the Analysis level. This connection is done by means of a *Deep Learning Control* module that loads and configures the models to carry out the inference. This module uses the ML.NET framework^52^ and the models exported to the ONNX format^53^ according to the specifications stored in XML files, which have the information required to define the CNN architecture and the trained parameters. The CNN Design Software has two modules: the *Custom Libraries* developed with PyTorch and FastAI to generate, design and train the models, and the *Exporter*, which exports the models to the Control and Image processing Software in the proper format.

The proposed design can be adapted to other scenarios simply by redefining the Project and the Analysis that fit the computer vision needs. The Analysis can be either a deterministic or a deep-learning approach. In the latter case, once the domain and the training of the model that best fits the scenario have been defined and prepared, the inference of the model with the appropriate export format can be applied immediately. Particularly, once the model has been trained, it has to be exported in ONNX format, and the XML file with the corresponding information has to be loaded. Then, the architecture is compiled in C# and the trained weights are assigned. The model that best fits the production requirements can be used, thus providing a flexible tool able to satisfy the changing industrial needs.

## Results and discussion

In this section, we present the different experiments that have been carried out to evaluate the proposed system in laboratory conditions and also in the real scenario.

All models used in this section are trained with an initial learning rate of 0.001, a first and second momentum of 0.09 and 0.999, an epsilon of $1{\mathrm {e}}-{05}$, and a weight decay of 0.01. Because neural network performance may be subject to minor random fluctuations, each training process has been repeated 5 times and only the best results are shown.

### Domain-specific dataset evaluation

To evaluate the five different domain-specific datasets presented in “Domain dataset preparation” section, the pre-trained ResNet18 has been used (as it is the simplest one) with 15 epochs and a batch size of 16 images. The obtained results, in terms of precision (*P*), recall (*R*), F-score (*F*), and accuracy (*A*), are presented in Table 3.

**Table 3:** Evaluation of the five different domain-specific datasets using a pre-trained ResNet18, where *P*, *R*, *F*, I, and *A* correspond to precision, recall, F-score, number of images used, and accuracy, respectively.

Label	*P*	*R*	*F*	*I*	*A*
**Product-based approach**					
**Whole bi-pack product**					
Reject	0.90	0.73	0.80	132	0.95
Accept	0.95	0.99	0.97	761	
**Bi-pack to single pack division**					
Reject	0.91	0.70	0.80	61	0.97
Accept	0.98	1.00	0.99	817	
**Sealing mask**					
Reject	0.90	0.78	0.84	132	0.96
Accept	0.96	0.99	0.97	761	
**Sealing-based approach**					
**Only sealing**					
Reject	1.00	0.94	0.97	32	0.98
Accept	0.97	1.00	0.99	78	
**Sealing mosaic**					
Reject	1.00	0.94	0.97	32	0.98
Accept	0.97	1.00	0.99	78	

Focusing on accuracy, although all of them are greater than or equal to 0.95, the best results are achieved with the sealing-based approach. Regarding the product-based approach, the best results are obtained with the *bi-pack to single pack division* dataset, followed by the *sealing mask* and the *whole bi-pack product* ones. In the worst-performing dataset, images contain information that is not relevant to our analysis, and hence this option is discarded. The best-performing dataset of the product-based approach presents a limitation, as not all images obtained from the division can be used. Therefore, this dataset is also discarded, leaving the *sealing mask* dataset as the best option for this approach. As for the sealing-based approach, the same results are obtained with the *only sealing* and *sealing mosaic* datasets. However, the latter is preferred since it uses images of $201 \times 201$ pixels, whereas the former uses images of $512 \times 512$ pixels obtained from a resizing process; that is, with the *sealing mosaic* dataset the speed increases and no information is lost due to resizing. Moreover, the *sealing mosaic* dataset is the only one in which the processed information has the same dimensions as the original images and all pixels contribute with relevant sealing information.

**Figure 8:**
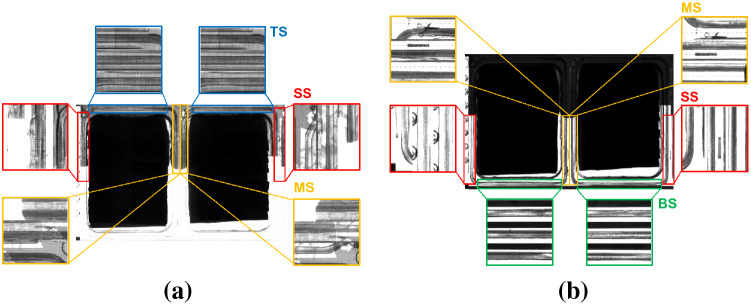
Images from the *sealing mosaic* dataset according to the different parts of the sealing that have been considered for the evaluation of the model in light (**a**) and dark (**b**) images (TS, BS, SS and MS correspond to top, bottom, side and middle sealing, respectively).

From this test, we conclude that the *sealing mosaic* is the best domain-specific dataset. Figure 8 shows some of its images according to the sealing zones.

### Performance of the CNN

To evaluate the performance of the CNN models, a complete domain-specific dataset has been created using the *sealing mosaic* strategy. Although the method aims to evaluate the whole product, to design the solution we decided to decompose the examination of the product into a set of phases, where each phase analyzes a part of the product. The phases have been defined considering the complexity of the region to be examined: from more to less complex regions. This decomposition has been carried out to better explore the results obtained and progressively advance to the final solution. Images are not removed when moving on to a new phase, but rather the dataset is expanded; that is, a new step includes new images in addition to the previous ones. A new phase has not been considered until the previous one has been duly resolved. First of all, the order of the phases seeks for the model to learn the most complex scenario first, thus ensuring the system’s viability, and then it seeks for the model to adjust this scenario for the following regions in the next steps, always on the basis of the complex case. With these considerations in mind, and to create the dataset, the sealing parts have been progressively evaluated in three phases, where each phase adds a new part of the sealing to the dataset, that is, a new part of the sealing is added to the previous phase. The first phase only takes into account the top sealing, as it is the most problematic due to factors related to brightness and serigraphy (non-transparent serigraphy). As a consequence of the product’s direction of advance, the top sealing is the place where more shadows and marks are generated because of its position with respect to the cameras and the machine itself. The bottom sealing is similar to the top sealing, as its position has the same characteristics; however, the film is transparent and it is easier to evaluate, so it is included in the second phase. Finally, all sealing zones are included in the third phase, as they are considered of less but equivalent complexity, and the effect of the film transparency has already been evaluated in previous phases. The accuracy would be similar if another order of phases were followed, since the final dataset would include the same images. However, the proposed order is able to give a slight importance to the most critical regions. The details of the three gradual datasets are presented in Table 4. In each phase, the model of the previous phase is re-trained taking into account the previous and new data. In the first phase, a model pre-trained with ImageNet has been considered, whereas the other phases have been re-trained with models obtained in the previous phase. Non-pre-trained and pre-trained ResNet18, ResNet50, Vgg19 and DenseNet161 have been evaluated with 60 epochs and a batch size of 32 images to determine which of them performs best. No testing dataset has been considered as testing will be carried out at the real installation using the best model obtained in this experiment.

**Table 4:** Details of the gradual datasets used to evaluate the performance of the CNN models.

Label $\rightarrow$	Accept	Reject
**Phase 1 dataset**		
Train (80.15%)	410	131
Validation (19.85%)	102	32
Total	512 (78.85%)	163 (24.15%)
**Phase 2 dataset**		
Train (80.00%)	582	171
Validation (20.00%)	144	44
Total	726 (77.15%)	215 (22.85%)
**Phase 3 dataset**		
Train (82.60%)	1987	991
Validation (17.40%)	439	189
Total	2426 (67.30%)	1180 (32.70%)

Table 5 presents the precision (*P*), recall (*R*), F-score (*F*) and number of validation images (I) for each phase, and Table 6 shows, also for each phase, the accuracy (*A*), training loss (*TL*) and validation loss (*VL*) of the best epoch obtained in the training process. Based on the results of the first phase, all models without pre-training are discarded, as pre-trained models obtain better results. Furthermore, ResNet18 is discarded in the second phase as ResNet50 achieves better results for both the reject and accept labels. Finally, the pre-trained DenseNet161 achieves the best results in the third phase with an average precision of 0.99 (0.98 for the reject label and 1.00 for the accept label), an average recall of 0.99 (0.99 for both labels), an average F-score of 0.99 (0.99 for both labels), an accuracy of 0.99, a train loss of 0.01, and a validation loss of 0.06. Pre-trained ResNet50 and Vgg19 also achieve good results, but they are not better than those of the pre-trained DenseNet161. Therefore, this evaluation concludes that the best model is the pre-trained DenseNet161. Note that our method achieves high accuracy values when compared with state-of-the-art methods (see Table 1).

**Table 5:** Precision (*P*), recall (*R*), F-score (*F*) and number of validation images (I) for each of the CNN models and for each phase of the training process (TS, BS, SS and MS correspond to top, bottom, side and middle sealing, respectively).

Phases $\rightarrow$	1: TS				2: TS and BS				3: TS, BS, SS, and MS			
Label	*P*	*R*	*F*	*I*	*P*	*R*	*F*	*I*	*P*	*R*	*F*	*I*
**ResNet18**												
Reject	1.00	0.84	0.92	32								
Accept	0.95	1.00	0.98	102								
**ResNet18 pre-trained**												
Reject	1.00	0.91	0.95	32	1.00	0.82	0.90	44				
Accept	0.97	1.00	0.99	102	0.95	1.00	0.97	144				
**ResNet50**												
Reject	1.00	0.75	0.86	32								
Accept	0.93	1.00	0.96	102								
**ResNet50 pre-trained**												
Reject	1.00	0.91	0.95	32	1.00	0.86	0.93	44	0.97	0.96	0.97	189
Accept	0.97	1.00	0.99	102	0.96	1.00	0.98	144	0.98	0.99	0.99	439
**Vgg19**												
Reject	0.92	0.75	0.83	32								
Accept	0.93	0.98	0.95	102								
**Vgg19 pre-trained**												
Reject	1.00	0.97	0.98	32	0.91	0.95	0.93	44	0.95	0.99	0.97	189
Accept	0.99	1.00	1.00	102	0.99	0.97	0.98	144	1.00	0.98	0.99	439
**DenseNet161**												
Reject	0.97	0.94	0.95	32								
Accept	0.98	0.99	0.99	102								
**DenseNet161 pre-trained**												
Reject	1.00	0.94	0.97	33	1.00	0.93	0.96	44	0.98	0.99	0.99	189
Accept	0.98	1.00	0.99	102	0.98	1.00	0.99	144	1.00	0.99	0.99	439

**Table 6:** Accuracy (*A*), train loss (*TL*) and validation loss (*VL*) of the best epoch obtained in the training process for each of the CNN models and for each phase (TS, BS, SS and MS correspond to top, bottom, side and middle sealing, respectively).

Phases $\rightarrow$	1: TS			2: TS and BS			3: TS, BS, SS and MS		
Model	*A*	*TL*	*VL*	*A*	*TL*	*VL*	*A*	*TL*	*VL*
**ResNet18**	0.96	0.10	0.17						
**ResNet18 pre-trained**	0.98	0.04	0.08	0.96	0.07	0.15			
**ResNet50**	0.94	0.07	0.52						
**ResNet50 pre-trained**	0.98	0.001	0.12	0.97	0.05	0.13	0.98	0.09	0.17
**Vgg19**	0.93	0.02	0.82						
**Vgg19 pre-trained**	0.99	0.03	0.08	0.97	0.12	0.12	0.98	0.03	0.10
**DensneNet161**	0.98	0.09	0.13						
**DensneNet161 pre-trained**	0.99	0.01	0.13	0.98	0.03	0.10	0.99	0.01	0.06

The pre-trained DenseNet161 confusion matrices are presented in Table 7. Each confusion matrix shows the number of images in each predicted (rows) and actual (columns) class, along with the false omission rate (FOR) and the false discovery rate (FDR). As intended, the FOR value is minimal in all cases, which ensures that faulty products are rejected. Similarly, the FDR value is also minimal and improves at each phase, which ensures that the desired system performance is achieved.

**Table 7:** Pre-trained DenseNet161 confusion matrices (rows and columns corresponding to predicted and actual classes, respectively), along with the false omission rate (*FOR*) and the false discovery rate (*FDR*), for the different phases of the testing (TS, BS, SS and MS correspond to top, bottom, side and middle sealing, respectively).

Confusion matrix	Reject	Accept	*FOR*	*FDR*
**Phase 1: TS**				
Reject	31	2	0	0.06
Accept	0	102		
**Phase 2: TS and BS**				
Reject	41	3	0	0.06
Accept	0	144		
**Phase 3: TS, BS, SS and MS**				
Reject	188	1	0.009	0.005
Accept	4	435		

### Testing in a real scenario

The phase-based process where different areas of the sealing are progressively evaluated was also applied to the tests in the real scenario. The sealing zones not considered by the model in the first two phases were not discarded, but evaluated via a computer-vision-based deterministic procedure developed with Halcon^32^ which uses evaluation filters to detect shape and gray features. In other words, the evaluation for the first two phases included both the deterministic procedure and the CNN model, as the CNN model was not yet complete, thus needing the supplementary deterministic procedure to evaluate all the sealing zones. The results obtained for each phase are presented in Table 8. Note that, throughout the testing process, adding parts of the sealing in the model leads to better results with no effect on the number of false positives. In fact, the false positives and negatives of the deterministic process eventually disappear, given that in the last phase only the CNN model, which is already complete, is used. In a 75-min test, the final solution achieved a false positive rate of 0.03%, a false negative rate of 0.03% and a rejection rate of 0.76%. As expected, the results are similar to those obtained in laboratory tests (see Table 7). Regarding *FOR* and *FDR* values, the former reaches the minimum value in all phases, while the latter decreases as the phase changes, reaching an acceptable final value.

**Table 8:** Total products considered (Total), accepted products (Accept), rejected products (Reject), test duration in minutes (M), true positives (*TP*), true negatives (TN), false positives of the deterministic procedure ($FP_D$), false positives of the CNN model ( *FP*), false negatives of the deterministic procedure ( $FN_D$), false negatives of the CNN model (*FN*), false omission rate (*FOR*) and false discovery rate (*FDR*) for each phase (P) of the real scenario test with the final pre-trained DenseNet161. Percentages have been computed from total production.

P	Total	Accept	Reject		M	TP	TN	$FP_D$		*FP*		$FN_D$		*FN*		*FOR*	*FDR*
**1**	3098	3066	32	*1.03%*	60	12	3065	13	*0.42%*	7	*0.23%*	1	*0.03%*	0	*0%*	0	0.90
**2**	2801	2755	46	*1.64%*	30	24	2755	16	*0.57%*	6	*0.21%*	0	*0%*	0	*0%*	0	0.48
**3**	2880	2858	22	*0.76%*	75	21	2857			1	*0.03%*			1	*0.03%*	$<0.001$	0.045

As a final test, as shown in Table 9, two more experiments were carried out, one considering an ideal production case without problems, and another one considering different errors in the thermoforming, sealing or cutting machines due to wear, bad configurations or external situations that affect production with a final impact on the sealing. The first experiment lasted 150 minutes and the proposed solution achieved a false positive rate of 0.06%, a false negative rate of 0% and a rejection rate of 0.64%. In the second experiment, which lasted 105 minutes, the results obtained reached a false positive rate of 0.30%, a false negative rate of 0.07% and a rejection rate of 5.09%. In both experiments, *FOR* and *FDR* values are suitable and similar to those obtained in previous experiments.

**Table 9:** Total products considered (Total), accepted products (Accept), rejected products (Reject), test duration in minutes (M), true positives (*TP*), true negatives (*TN*), false positives of the CNN model (FP), false negatives of the CNN model (*FN*), false omission rate (*FOR*) and false discovery rate (*FDR*) obtained with the pre-trained DenseNet161 in the real scenario considering two production situations: one without problems, and another one with problems. Percentages have been computed from total production.

Production	Total	Accept	Reject		M	TP	TN	*FP*		*FN*		*FOR*	*FDR*
**Without problems**	6576	6534	42	*0.64%*	150	38	6534	4	*0.06%*	0	*0%*	0	0.095
**With problems**	4304	4085	219	*5.09%*	105	206	4082	13	*0.30%*	3	*0.07%*	0.001	0.059

These results conclude that the CNN-based approach improves the efficiency of the quality control process with optimal results in all situations, not only the ideal one. The proposed system achieves false positive rates that range from 0.03 to 0.30% and false negative rates that range from 0 to 0.07%. It has a rejection rate between 0.64 and 5.09% of production and it will detect at least 99.93% of sealing defects that occur in any production.

## Conclusions and future work

In this paper, a CNN-based approach designed to perform the quality control of sealing and closure of thermoforming packaging has been presented. Based on a practical case in a real industrial scenario, the main components that have to be considered to design a CNN-based solution have been described in detail. In addition, different CNN input datasets and models have been evaluated to determine which one best fits the situation under study. The experiments have shown that the pre-trained DenseNet161 obtains optimal results, and that the proposed CNN-based approach improves the efficiency and security of the quality control process. Because CNN solutions have to be defined according to the features of the scenario, and they have to adapt to computer vision systems, the modular design that has been proposed allows the approach to fit similar scenarios.

Our future work will be centered on the extension of the proposed solution to support other types of thermoforming packages containing non-food products. Our aim is to evaluate the solution in these new scenarios in order to provide a more global solution.
